# PD-1 Expression by Lymph Node and Intratumoral Regulatory T Cells Is Associated with Lymph Node Metastasis in Pancreatic Cancer

**DOI:** 10.3390/cancers12102756

**Published:** 2020-09-24

**Authors:** Adrian M. Seifert, Annabel Eymer, Max Heiduk, Rebekka Wehner, Antje Tunger, Janusz von Renesse, Rahel Decker, Daniela E. Aust, Thilo Welsch, Christoph Reissfelder, Jürgen Weitz, Marc Schmitz, Lena Seifert

**Affiliations:** 1 Department of Visceral, Thoracic and Vascular Surgery, Medical Faculty, University Hospital Carl Gustav Carus, TU Dresden, 01307 Dresden, Germany; max.heiduk@ukdd.de (M.H.); janusz.vonrenesse@ukdd.de (J.v.R.); rahel.decker@ukdd.de (R.D.); thilo.welsch@ukdd.de (T.W.); juergen.weitz@ukdd.de (J.W.); lena.seifert@ukdd.de (L.S.); 2 German Cancer Consortium (DKTK), Partner Site Dresden, German Cancer Research Center (DKFZ), 69120 Heidelberg, Germany; marc.schmitz@tu-dresden.de; 3 Institute of Immunology, Medical Faculty Carl Gustav Carus, TU Dresden, 01307 Dresden, Germany; annabel.eymer@me.com (A.E.); rebekka.wehner2@mailbox.tu-dresden.de (R.W.); antje.tunger@ukdd.de (A.T.); 4 National Center for Tumor Diseases (NCT), Partner Site Dresden, German Cancer Research Center (DKFZ), 69120 Heidelberg, Germany; 5 National Center for Tumor Diseases (NCT), University Hospital Carl Gustav Carus, TU Dresden, 01307 Dresden, Germany; 6 Department of Pathology, University Hospital Carl Gustav Carus, Medical Faculty, University of Dresden, 01307 Dresden, Germany; daniela.aust@ukdd.de; 7 NCT Biobank Dresden, University Hospital Carl Gustav Carus, Technische Universität Dresden, 01307 Dresden, Germany; 8 Department of Surgery, Universitätsmedizin Mannheim, Medical Faculty Mannheim, Heidelberg University, 68167 Mannheim, Germany; christoph.reissfelder@umm.de

**Keywords:** pancreatic cancer, tumor-draining lymph nodes, T cells, Treg cells, PD-1

## Abstract

**Simple Summary:**

Pancreatic cancer is a devastating disease and among the most immune-resistant tumor types. Single-agent immunotherapy has not demonstrated clinical benefits in pancreatic cancer patients, and combinational therapies targeting multiple mechanisms of immunosuppression are likely needed. T cell activation in lymph nodes is required for the efficacy of immunotherapy. Here, we phenotypically and functionally analyze T cells from tumor-draining lymph nodes, blood and tumors from patients with pancreatic cancer to decipher unknown immunosuppressive mechanisms and to identify potential immunotherapeutic targets.

**Abstract:**

Pancreatic ductal adenocarcinoma (PDAC) is characterized by a mostly immunosuppressive microenvironment. Tumor-draining lymph nodes (TDLN) are a major site for priming of tumor-reactive T cells and also tumor metastasis. However, the phenotype and function of T cells in TDLNs from PDAC patients is unknown. In this study, lymph nodes from the pancreatic head (PH), the hepatoduodenal ligament (HDL) and the interaortocaval (IAC) region were obtained from 25 patients with adenocarcinoma of the pancreatic head. Additionally, tumors and matched blood were analyzed from 16 PDAC patients. Using multicolor flow cytometry, we performed a comprehensive analysis of T cells. CD4^+^ T cells were the predominant T cell subset in PDAC-draining lymph nodes. Overall, lymph node CD4^+^ and CD8^+^ T cells had a similar degree of activation, as measured by CD69, inducible T cell co-stimulator (ICOS) and CD137 (4-1BB) expression and interferon-γ (IFNγ) secretion. Expression of the inhibitory receptor programmed death 1 (PD-1) by lymph node and tumor-infiltrating regulatory T cells (Tregs) correlated with lymph node metastasis. Collectively, Treg cells and PD-1 are two relevant components of the immunosuppressive network in PDAC-draining lymph nodes and may be particularly attractive targets for combinatorial immunotherapeutic strategies in selected patients with node-positive PDAC.

## 1. Introduction

Pancreatic ductal adenocarcinoma (PDAC) is a devastating disease with fewer than 9% of patients surviving five years [1]. Lymph node (LN) metastases occur in approximately two-thirds of patients with resectable PDAC and are associated with poor prognosis [2,3]. In multiple solid tumors, including kidney, bladder and lung cancer, as well as melanoma, immunotherapeutic strategies substantially improved the overall survival. So far, the use of a single-agent immune checkpoint blockade has not demonstrated clinical benefits, however a very limited number of PDAC patients has been enrolled in these studies [4,5,6]. Notably, T cell activation in regional lymph nodes is required for the efficacy of inhibitory receptor blockade [7].

There is relevant data suggesting a heterogeneity with regard to the immunogenicity of PDAC [8]. T cells are the major immune cell type infiltrating PDAC, and the majority of resectable PDAC samples display intermediate to high levels of T cell infiltration [9,10,11]. Among these, CD4^+^ T cells, and especially conventional CD4^+^ T cells (Tconv), are the predominant T cell subset [12]. Further, spatial distribution of cytotoxic CD8^+^ T cells in proximity to tumor cells correlates with improved overall survival in pancreatic cancer [13]. An increased frequency of tumor-infiltrating regulatory T cells (Tregs) is associated with poor prognosis and Treg density further correlates with lymph node metastasis in PDAC [14,15,16].

The programmed death 1 (PD-1) and programmed death ligand 1 (PD-L1) axis has become a central target of immunotherapeutic approaches. PD-1 is an inhibitory receptor that is upregulated after T-cell activation and remains elevated with antigen persistence. Ligation of PD-1, through its ligands PD-L1 (B7-H1, CD274) and PD-L2 (B7-DC, CD273), delimits immunogenic responses. PD-L1 is expressed on immune and tumor cells and is associated with worse outcomes in PDAC [17,18].

The state of T cell activation and exhaustion in tumor-draining lymph nodes (TDLNs) in human PDAC is unknown. In this study, we analyzed freshly isolated immune cells from several lymph nodes, blood and tumor specimens of PDAC patients.

## 2. Results

### 2.1. CD4^+^ T Cells Are the Predominant T Cell Subset in PDAC-Draining Lymph Nodes

**Figure 1:**
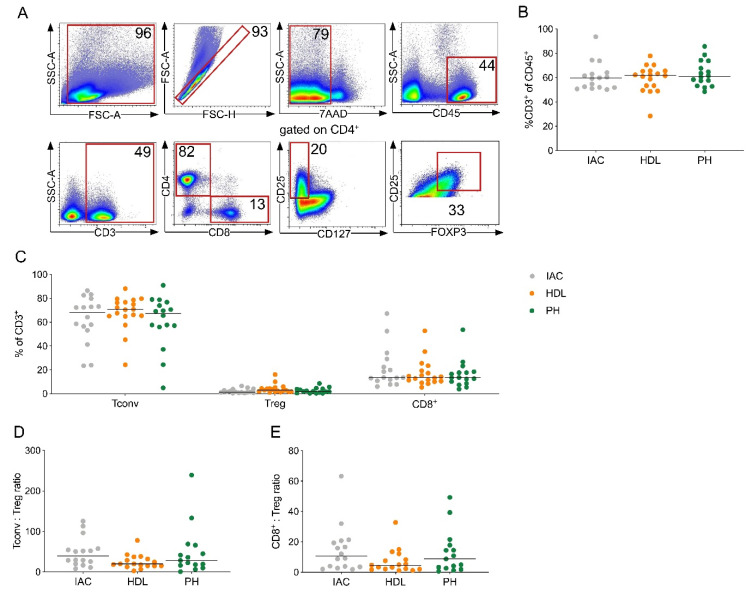
CD4^+^ T cells are the predominant T cell subset in pancreatic ductal adenocarcinoma (PDAC)-draining lymph nodes. (**A**) Representative flow cytometric gating strategy for the identification of T cells. Number indicates percentage of population per gate. SSC, side scatter; FSC, forward scatter. (**B**) Quantification of CD3^+^ T cells among all leucocytes (CD45^+^). (**C**) CD4^+^ Tconv cells (Tconv; CD3^+^CD4^+^’not Treg’), regulatory T cells (Treg; CD3^+^CD4^+^CD8^-^CD25^+^CD127^-^FOXP3^+^) and CD8^+^ T cells (CD8^+^; CD3^+^CD4^-^CD8^+^) as a percentage of CD3^+^ T cells in lymph nodes of the indicated location from patients with PDAC. (**D**) Ratio of CD4^+^ Tconv to Treg and (**E**) CD8^+^ T cells to Treg in lymph nodes. IAC, interaortocaval: lymph node around the abdominal aorta; HDL, hepatoduodenal ligament: lymph node along the hepatic artery and bile duct; PH, pancreatic head: lymph node from the posterior aspect of the pancreatic head. Each point represents data from one patient. Data, median. One-way ANOVA.

To determine the composition of T cell subsets in lymph nodes from PDAC patients, we performed flow cytometry on lymph nodes from three different locations, namely the interaortocaval (IAC), hepatoduodenal ligament (HDL) and pancreatic head (PH) lymph node region. All lymph nodes were freshly obtained and analyzed from a total of 25 PDAC patients undergoing surgery at our institution (Table S1). Representative dot plots for T cell gating are shown (Figure 1A).

The percentages of CD3^+^ T cells among leukocytes was similar between lymph node regions (Figure 1B). Overall, CD4^+^ Tconv cells were the predominant T cell population in lymph nodes (Figure 1C). Further, the CD4^+^ Tconv to Treg as well as the CD8^+^ T cell to Treg ratio was similar between lymph node locations (Figure 1D,E). These data suggest that the frequency of T cell subsets is unrelated to the distance of lymph nodes from the primary tumor.

### 2.2. Central and Transitionally Memory T Cells Constitute the Predominant T Cell Differentiation Stages in PDAC-Draining Lymph Nodes

**Figure 2:**
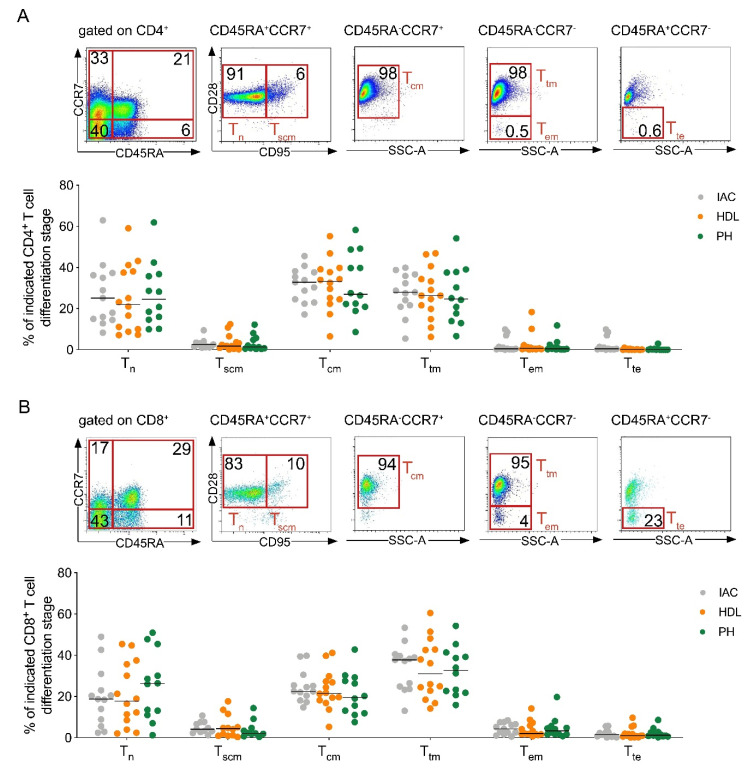
Central and transitionally memory T cells constitute the predominant T cell differentiation stages in PDAC-draining lymph nodes. (**A**) Representative flow plots (top) and frequency (below) of T cell differentiation stage in lymph nodes of the indicated location for CD4^+^ and (**B**) CD8^+^ T cells. Tn, naïve (CD45RA^+^CCR7^+^CD28^+^CD95^-^); Tscm, stem cell memory (CD45RA^+^CCR7^+^CD28^+^CD95^+^); Tcm, central memory (CD45RA^-^CCR7^+^CD28^+^); Ttm, transitionally memory (CD45RA^-^CCR7^-^CD28^+^); Tem, effector memory (CD45RA^-^CCR7^-^CD28^-^); Tte, terminal effector (CD45RA^+^CCR7^-^CD28^-^). IAC, interaortocaval; HDL, hepatoduodenal ligament; PH, pancreatic head. Each point represents data from one patient. Data, median. One-way ANOVA.

Next, we analyzed the differentiation stages of T cells in PDAC-draining lymph nodes. Following antigenic stimulation, naive T cells generate multiple subsets of memory T cells with different phenotypic and functional properties [19]. The expression of CD45RA, C-C chemokine receptor 7 (CCR7), CD28 and CD95 was determined by flow cytometry (Figure 2A,B).

Overall, central memory CD4^+^ T cells (Figure 2A) and transitionally memory CD8^+^ T cells (Figure 2B) were the predominant T cell subsets in PDAC-draining lymph nodes. Notably, naive T cells also represented a significant subgroup in CD4^+^ and CD8^+^ T cells (27% and 19.8%, respectively). The distribution of memory T cells within the CD4^+^ and CD8^+^ T cell subset was similar between lymph node regions.

### 2.3. Lymph Node CD4^+^ and CD8^+^ T Cells Display a Similar Degree of Activation Independent of Distance from the Tumor

**Figure 3:**
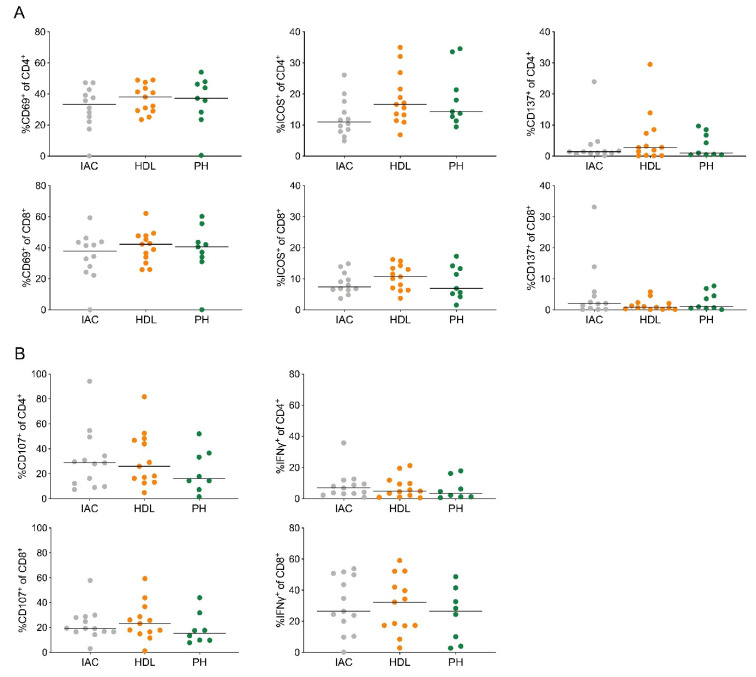
Lymph node CD4^+^ and CD8^+^ T cells display a similar degree of activation independent of distance from the tumor. (**A**) Quantification of the expression of the activation markers CD69 (left), ICOS (middle) and CD137 (right) on CD4^+^ (top) and CD8^+^ T cells (bottom). (**B**) Quantification of CD107a (left) expression and IFNγ (right) secretion by stimulated CD4^+^ (top) and CD8^+^ T cells (bottom). IAC, interaortocaval; HDL, hepatoduodenal ligament; PH, pancreatic head. Each point represents data from one patient. Data, median. One-way ANOVA.

To determine differences in T cell activation in lymph node CD4^+^ and CD8^+^ T cells from PDAC patients, we stained for the activation markers CD69, inducible T cell co-stimulator (ICOS) and CD137 (4-1BB). Approximately 40% of CD4^+^ and CD8^+^ T cells expressed CD69 (Figure 3A).

CD4^+^ T cells showed higher levels of ICOS (18.8%) compared to CD8^+^ T cells (8.8%, Figure 3A). The expression of CD137 was generally low on CD4^+^ (3.6%) and CD8^+^ T cells (2.8%, Figure 3A). We next analyzed CD107a expression, a surrogate marker for cytotoxic T cell degranulation, and interferon-γ (IFNγ) secretion (Figure 3B). CD8^+^ T cells showed higher IFNγ secretion (24%) compared to CD4^+^ T cells (6.3%), but no difference in CD107a expression (22.6 and 31%, respectively, Figure 3B). Collectively, the degree of T cell activation was similar in lymph nodes from different regions and unrelated to distance from the primary tumor.

### 2.4. Treg Cells in PDAC-Draining Lymph Nodes Express PD-1 and PD-L1

**Figure 4:**
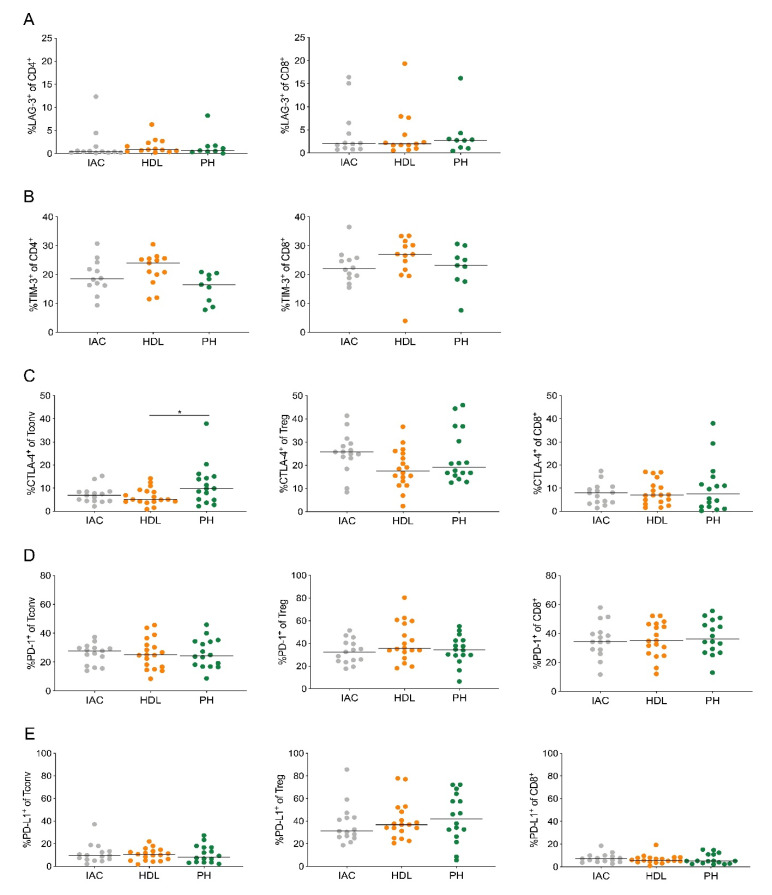
Treg cells in PDAC-draining lymph nodes express PD-1 and PD-L1. (**A**) Quantification of the expression of LAG-3 and (**B**) TIM-3 on CD4^+^ (left) and CD8^+^ T cells (right). (**C**) Quantification of the expression of CTLA-4, (**D**) PD-1 and (**E**) PD-L1 on CD4^+^ Tconv cells (Tconv; left), regulatory T cells (Treg; middle) and CD8^+^ T cells (CD8^+^; right). IAC, interaortocaval; HDL, hepatoduodenal ligament; PH, pancreatic head. Each point represents data from one patient. Data, median. One-way ANOVA. * *p* < 0.05.

Antigen-specific and exhausted T cells typically express inhibitory receptors, including lymphocyte activation gene 3 (LAG-3), T cell immunoglobulin and mucin-domain containing-3 (TIM-3), cytotoxic T-lymphocyte-associated protein 4 (CTLA-4) and PD-1 [20,21]. Therefore, we analyzed the expression of these inhibitory receptors and also PD-L1 on lymph node T cells. CD4^+^ and CD8^+^ T cells both displayed very low LAG-3 expression (1.6% and 3.8%, respectively; Figure 4A).

However, approximately 20% of CD4^+^ and CD8^+^ T cells expressed TIM-3 (Figure 4B). Treg cells showed higher CTLA-4 expression (26.3%) compared to CD4^+^ Tconv (11.5%) and CD8^+^ T cells (10.2%; Figure 4C). Notably, CD4^+^ Tconv, Treg and CD8^+^ T cells highly expressed PD-1 (25.9%, 29.6% and 37.6%, respectively; Figure 4D). PD-L1 was mostly expressed by Treg cells (53.6%; Figure 4E). Especially, Treg cells from the pancreatic head lymph node displayed high PD-L1 expression. These data suggest that the PD-1/PD-L1 axis contributes to the immunosuppressive network in PDAC-draining lymph nodes.

### 2.5. PD-1-Expressing Lymph Node T Cells Are Associated with Node-Positive PDAC

**Figure 5:**
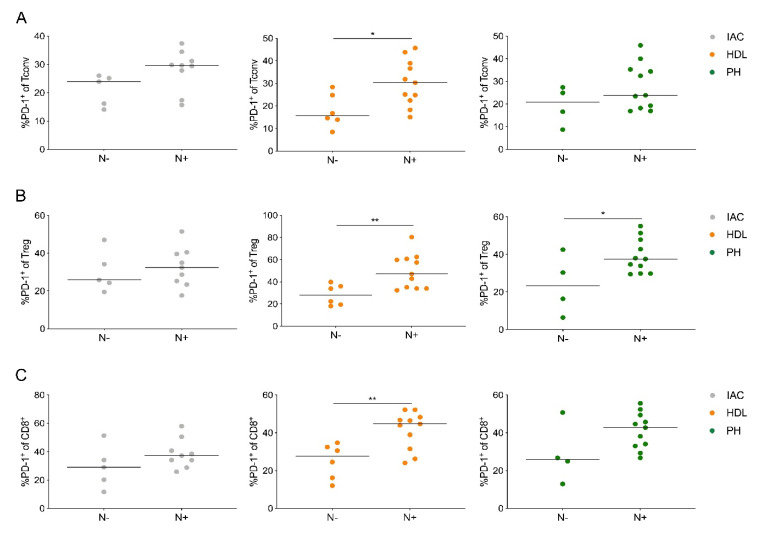
PD-1-expressing lymph node T cells are associated with node-positive PDAC. (**A**) Quantification of the expression of PD-1 on CD4^+^ Tconv cells (Tconv), (**B**) regulatory T cells (Treg) and **(C)** CD8^+^ T cells (CD8^+^) based on nodal stage (N-, negative; N+, positive). IAC, interaortocaval; HDL, hepatoduodenal ligament; PH, pancreatic head. Each point represents data from one patient. Data, median. Unpaired *t*-test. * *p* < 0.05, ** *p* ≤ 0.01.

Next, we analyzed the relationship of inhibitory receptor expression in T cells with tumor stage. Despite no relevant association of PD-1 expression with tumor size (data not shown), patients with lymph node metastasis (N+) had significantly higher PD-1 expression on CD4^+^ Tconv, Treg cells and CD8^+^ T cells compared to patients without lymph node metastasis (N-; Figure 5A–C).

Especially in the lymph node from the hepatoduodenal ligament PD-1, expression by all three T cell populations was associated with lymph node metastasis. Collectively, PD-1 expression by lymph node T cells correlates with nodal disease and may be involved in the metastatic process into regional lymph nodes in PDAC.

### 2.6. PD-1 Expression by Intratumoral Treg Cells Correlates with Node-Positive PDAC

**Figure 6:**
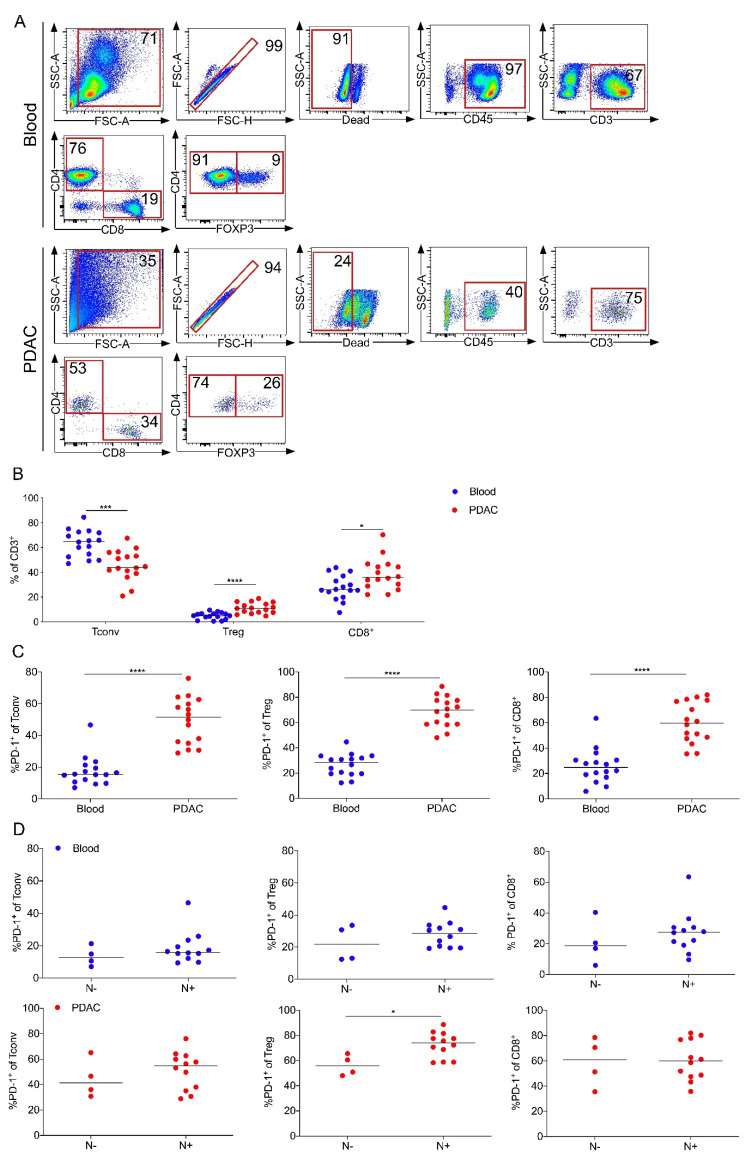
PD-1 expression by intratumoral Treg cells correlates with node-positive PDAC. (**A**) Representative flow cytometric gating strategy for the identification of T cells in blood (top) and PDAC (bottom). Number indicates percentage of population per gate. SSC, side scatter; FSC, forward scatter. (**B**) CD4^+^ Tconv cells (Tconv; CD3^+^CD4^+^CD8^-^FOXP3^-^), regulatory T cells (Treg; CD3^+^CD4^+^CD8^-^FOXP3^+^) and CD8^+^ T cells (CD8^+^; CD3^+^CD4^-^CD8^+^) as a percentage of CD3^+^ T cells in blood and tumor from 16 patients with PDAC. (**C**) Percentages of PD-1 expression of indicated T cell subset in blood and PDAC. (**D**) Quantification of the expression of PD-1 on blood (top) and tumor-infiltrating T cells (PDAC, bottom) based on nodal stage (N-, negative; N+, positive). Each point represents data from one patient. Data, median. Unpaired *t*-test. **** *p* ≤ 0.0001, *** *p* ≤ 0.001, * *p* < 0.05.

To further elucidate the role of PD-1 expression by T cells in PDAC patients, we analyzed tumors and matched blood samples from a separate cohort of 16 PDAC patients (Table S2) and determined PD-1 expression using flow cytometry. Representative dot plots for T cell gating are shown (Figure 6A).

The frequency of Treg cells and CD8^+^ T cells was significantly increased in PDAC compared to matched blood samples (Figure 6B). Further, all intratumoral T cells displayed higher PD-1 expression compared to their circulating counterpart (Figure 6C). T cell frequencies were not associated with lymph node metastasis (data not shown), whereas PD-1 expression by intratumoral Treg cells correlated with node-positive PDAC (N+, Figure 6D). Thus, consistent with our previous observation from TDLNs, PD-1 expression by intratumoral Treg cells also correlated with lymph node metastasis.

## 3. Discussion

The 5-year survival for microscopically margin-negative (R0)-resected PDAC patients with lymph node metastasis ranges from 10 to 15%, as opposed to 37% for patients without lymphatic tumor cell dissemination [2]. TDLNs are not only the preferential site of initial tumor metastasis, but also the primary site of tumor antigen presentation, immune activation and regulation. There is substantial evidence that immune modulation occurs not only in the tumor, but also in TDLNs, which may facilitate lymph node metastasis [22].

In this study, we analyzed T cells from different lymph node locations relative to the primary tumor in the pancreatic head. According to the probability of lymph node metastasis, we chose lymph nodes from the posterior aspect of the pancreatic head (pancreatic head, PH), along the hepatic artery and bile duct (hepatoduodenal ligament, HDL) and around the abdominal aorta (interaortocaval, IAC) for analysis (54.3%, 16.1%, and 12.5%, respectively) [3]. Notably, interaortocaval lymph node involvement is considered as distant metastasis and tumors are staged UICC IV accordingly [23].

Our comprehensive flow cytometric analysis indicated that T cells in PDAC-draining lymph nodes have a similar frequency, differentiation stage and phenotype independent of proximity to the tumor. However, we provide evidence for a relevant role of PD-1-expression for lymph node metastasis in PDAC. Our findings suggest that the presence of PD-1^+^ T cells either occurs before lymphatic tumor cell spread and potentially facilitates tumor cell infiltration, or that Treg cells in TDLNs upregulate PD-1 in response to their interaction with tumor cells. Generally, PD-1 expression has been demonstrated to identify T cells that recognize tumor-specific proteins [24,25]. Our data indicate a relevant role for PD-1 in the immune suppressive network of PDAC-draining lymph nodes.

Additionally, we observed that PD-1 expression by intratumoral Treg cells correlated with lymph node metastasis. Whether Treg cells contribute directly to conditioning of premetastatic niches warrants further investigation. Notably, in a recent study, intratumoral Treg cell density correlated with lymphatic metastasis [15]. PDAC cells express high levels of C-C chemokine ligand 5 (CCL5) to recruit Treg cells through C-C chemokine receptor 5 (CCR5) [26]. In cervical cancer patients, a PD-L1-expressing antigen-presenting cell (APC) subset in metastatic lymph nodes correlated with an increased frequency of Treg cells [27]. Colorectal cancer patients with lymph node metastasis had an increased frequency of Treg cells, which was associated with impaired CD8^+^ T cell function [28]. However, in our study, Treg cell frequency was not associated with lymph node metastasis. The frequency of CD10^+^ pancreatic stellate cells in PDAC correlated with nodal metastasis and shorter survival [29]. Additionally, IL-4-expressing basophils have been shown to be enriched in PDAC-draining lymph nodes and the percentage of basophils in the TDLNs was identified as an independent prognostic factor for worse survival after surgery [30]. The presence of PD-1^+^ Treg cells may be useful as a predictive marker for patient survival, but unlikely a stronger predictive factor than lymph node metastasis itself.

Multiple preclinical murine models show that Treg cells are important for the establishment of metastatic sites following dissemination of tumor cells [31]. For example, the receptor activator of nuclear factor kappa-B ligand (RANKL) on Treg cells directly promoted pulmonary metastasis in breast cancer [32]. In a murine PDAC model, Treg cells restrained the function of dendritic cells (DCs) by suppressing the expression of costimulatory ligands necessary for CD8^+^ T cell activation [33]. Further investigations into the mechanisms of Treg recruitment and their role at metastatic sites are warranted.

Further, neoadjuvant therapy was associated with a reduction of suppressive immune cells, including Treg cells and myeloid-derived suppressor cells (MDSCs) in PDAC [34]. Long-term treatment with gemcitabine has been shown to lead to extensive reprogramming of the pancreatic cancer microenvironment, sensitizing murine PDAC to immunotherapy [35]. In the setting of TGFβ signaling deficiency, gemcitabine and PD-1 blockade reduced tumor growth mediated through CD8^+^ T cells. Agonist anti-CD40 therapy with chemotherapy reversed the complete resistance of murine pancreatic tumors to PD-1 and CTLA-4 blockade [36]. Endogenous tumor-reactive T cells are present within the human PDAC tumor microenvironment and can be reactivated by combined blockade of PD-1 and C-X-C chemokine receptor 4 (CXCR4) [37]. These preclinical observations argue against the currently accepted paradigm that PDAC does not respond to immunotherapy due to a lack of immunogenicity or generation of tumor-reactive T cells.

## 4. Materials and Methods

### 4.1. Patient Samples

Blood, tumor and lymph node specimens were obtained from patients with PDAC, who underwent surgery at our institution. All patients consented to a protocol approved by the Ethics Committee of the TU Dresden (No EK446112017). Lymph nodes from the posterior aspect of the pancreatic head, along the hepatic artery and bile duct, and around the abdominal aorta were identified and divided [38]. One part was formalin-fixed, paraffin-embedded and a serial section was stained with H&E for histologic evaluation, while the remaining specimen was subjected to mechanical dissociation to obtain single-cell suspensions. Blood and tumors were processed as previously described [39]. Cells were analyzed with flow cytometry. Only lymph nodes with a sufficient cell number for each multicolor flow cytometry panel were used. The clinical stages of tumors were determined according to the tumor-node-metastasis (TNM) classification system by the Union for International Cancer Control (UICC; Edition 8). Patient characteristics are shown in Tables S1 and S2.

### 4.2. Flow Cytometry and Antibodies

Single-cell suspensions for flow cytometry were prepared and samples were stained with monoclonal antibodies directed against CD45 (HI30), CD3 (SK7 or UCHT1), CD4 (RPA-T4), CD8 (RUO), CD25 (M-A251), CD27 (M-T271), CD28 (CD28.2), CD45RA (H100), CD45RO (UCHL1), CD69 (L78), CD95 (DX2), CD107a (H4A3), CD127 (HIL-7R-M21), CD197 (3D12), CD274 (MIH1), CD19 (HIB19), FOXP3 (259D/C7), IFN-γ (25723.11, all BD Biosciences), CD123 (6H6), CD152 (L3D10), CD279 (EH12.2H7), ICOS (C398.4A), TIM-3 (F38-2E2, all BioLegend; San Diego, USA), and LAG-3 (R&D Systems; Minneapolis, USA) according to the manufacturer’s protocol. FOXP3 was stained using the human FOXP3 Buffer Set (BD Biosciences; Franklin Lakes, USA) according to the manufacturer’s protocol. For intracellular cytokine staining, cells were stimulated with phorbol 12-myristate 13-acetate (PMA, 50 ng/mL) and ionomycin (750 ng/mL) for 4 hours at 37 °C, 5% CO2 in the presence of 1 mg/mL brefeldin A (BD Biosciences; Franklin Lakes, USA). Surface staining was performed, and cells were fixed and permeabilized with the BD Cytofix/Cytoperm Kit and stained for CD107a and IFN-γ. Flow cytometry was carried out on the Aria flow cytometer (BD Biosciences; Franklin Lakes, USA). Data were analyzed using FlowLogic 700.2a (Inivai^TM^ Technologies, Mentone, Australia).

### 4.3. Statistical Analysis

Data are shown as median ± SEM. Unpaired Student’s *t*-test or one-way ANOVA comparisons were performed as applicable. GraphPad Prism 8.0 (GraphPad Software, La Jolla, CA) was used. *p* ≤ 0.05 was considered significant.

## 5. Conclusions

In this study, we broadly characterized the composition and phenotype of T cells within TDLNs in human PDAC. Our data identified PD-1 and Treg cells as relevant elements of the immunosuppressive network contributing to lymphatic tumor cell spread in human PDAC. Both components are potential immunotherapeutic targets for selected PDAC patients with lymph node metastasis.

## Supplementary Materials

The following are available online at https://www.mdpi.com/2072-6694/12/10/2756/s1, Table S1: Clinicopathologic characteristics of patients in the lymph node cohort, Table S2: Clinicopathologic characteristics of patients in the blood and tumor cohort.
