# Supplementary material for: PD-1 Expression by Lymph Node and Intratumoral Regulatory T Cells Is Associated with Lymph Node Metastasis in Pancreatic Cancer
Source: Cancers (Basel). 2020 Sep 24;12(10):2756. doi: 10.3390/cancers12102756 (PMC7599971; doi:10.3390/cancers12102756)
Supplement: Supplementary file 1 [file cancers-12-02756-s001.pdf]

Supplementary Materials

Table S1. Clinicopathologic characteristics of patients in the lymph node cohort.

	**n = 25**<br>**n (%)**
Age	
Median (range)	66 (45–81)
Gender	
Male	17 (68)
Female	8 (32)
pT Stage	
1	0 (0)
2	12 (48)
3	11 (44)
4	1 (4)
Unknown	1 (4)
pN Stage	
0	7 (28)
1	12 (48)
2	5 (20)
Unknown	1 (4)
pM Stage	
0	20 (80)
1	5 (20)
UICC Stage	
I	3 (12)
II	14 (56)
III	3 (12)
IV	5 (20)
Neoadjuvant Treatment	
Yes	6 (24)
FOLFIRONOX	4 (16)
Gemcitabine/Nab-paclitaxel	2 (8)
No	19 (76)

Table S2. Clinicopathologic characteristics of patients in the blood and tumor cohort.

	**n = 16**<br>**n (%)**
Age, y	
Median (range)	69 (56-82)
Gender	
Male	6 (38)
Female	10 (62)
pT Stage	
1	0 (0)
2	8 (50)
3	7 (44)
4	1 (6)
pN Stage	
0	4 (25)
1	5 (31)
2	7 (44)
pM Stage	
0	12 (75)
1	4 (25)
UICC Stage	
I	2 (13)
II	5 (31)
III	5 (31)
IV	4 (25)
Neoadjuvant Treatment	
Yes	7 (44)
FOLFIRONOX	4 (25)
Gemcitabine/Nab-paclitaxel	3 (19)
No	9 (56)
